# Quantifying the Digital Divide: Associations of Broadband Internet with Tele-mental Health Access Before and During the COVID-19 Pandemic

**DOI:** 10.1007/s11606-023-08120-8

**Published:** 2023-06-20

**Authors:** Amy M. J. O’Shea, M. Bryant Howren, Kailey Mulligan, Bjarni Haraldsson, Ariana Shahnazi, Peter J. Kaboli

**Affiliations:** 1 Veterans Rural Health Resource Center-Iowa City, VA Office of Rural Health, Iowa City VA Healthcare System, Iowa City, IA USA; 2 Center for Access and Delivery Research and Evaluation (CADRE), Iowa City VA Healthcare System, Iowa City, IA USA; 3 Department of Internal Medicine, University of Iowa Carver College of Medicine, Iowa City, IA USA; 4 Department of Behavioral Sciences and Social Medicine, Florida State University College of Medicine, Tallahassee, FL USA; 5 Florida Blue Center for Rural Health Research & Policy, Florida State University College of Medicine, Tallahassee, FL USA; 6 Department of Biostatistics, University of Iowa College of Public Health, Iowa City, IA USA

**Keywords:** telemedicine, COVID-19, mental health, internet access, veterans

## Abstract

**Background:**

During the COVID-19 pandemic, telemedicine quickly expanded. Broadband speeds may impact equitable access to video-based mental health (MH) services.

**Objective:**

To identify access disparities in Veterans Health Administration (VHA) MH services based on broadband speed availability.

**Design:**

Instrumental variable difference-in-differences study using administrative data to identify MH visits prior to (October 1, 2015–February 28, 2020) and after COVID-19 pandemic onset (March 1, 2020–December 31, 2021) among 1176 VHA MH clinics. The exposure is broadband download and upload speeds categorized as inadequate (download  ≤25 Megabits per second - Mbps; upload  ≤3 Mbps), adequate (download  ≥25 Mbps and  <100 Mbps; upload  ≥5 Mbps and  <100 Mbps), or optimal (download and upload  ≥100/100 Mbps) based on data reported to the Federal Communications Commission at the census block and spatially merged to each veteran’s residential address.

**Participants:**

All veterans receiving VHA MH services during study period.

**Main Measures:**

MH visits were categorized as in-person or virtual (i.e., telephone or video). By patient, MH visits were counted quarterly by broadband category. Poisson models with Huber-White robust errors clustered at the census block estimated the association between a patient’s broadband speed category and quarterly MH visit count by visit type, adjusted for patient demographics, residential rurality, and area deprivation index.

**Key Results:**

Over the 6-year study period, 3,659,699 unique veterans were seen. Adjusted regression analyses estimated the change after pandemic onset versus pre-pandemic in patients’ quarterly MH visit count; patients living in census blocks with optimal versus inadequate broadband increased video visit use (incidence rate ratio (IRR) = 1.52, 95% CI = 1.45–1.59; *P* < 0.001) and decreased in-person visits (IRR = 0.92, 95% CI = 0.90–0.94; *P* < 0.001).

**Conclusions:**

This study found patients with optimal versus inadequate broadband availability had more video-based and fewer in-person MH visits after pandemic onset, suggesting broadband availability is an important determinant of access-to-care during public health emergencies requiring remote care.

**Supplementary Information:**

The online version contains supplementary material available at 10.1007/s11606-023-08120-8.

## INTRODUCTION

The COVID-19 pandemic increased telemedicine use in the Veterans Health Administration (VHA) to protect patients and clinicians.^1,2^ Although most virtual care was delivered by telephone, video visits also increased, a substantial portion of which was dedicated to mental health (MH) services.^2–4^ Worldwide increases in MH morbidity have been documented since pandemic onset and are expected to worsen due to the physical, emotional, social, and economic tolls of COVID-19.^5–7^ Tele-MH care is important when in-person care is limited and is increasingly important to maintain and improve access to MH care in the post-COVID era.

In the last decade, technology advancements allowed consistent delivery of face-to-face (i.e., synchronous, clinician-to-patient) care via streaming video. Further, the equivalence of tele-MH services to in-person care is well-established across outcomes, including symptom improvement, adherence, and patient satisfaction.^4,8,9^ The VHA has long been a video telemedicine pioneer, including tele-MH services, which helped veterans rapidly transition to telemedicine.^4,10,11^ Patient-level barriers to tele-MH adoption remain. Predictors of reduced adoption include age, income, education, rurality, and establishing care as a new patient.^12,13^ The availability of adequate broadband Internet speeds needed for streaming video may also be a critical factor.

For this study, broadband refers to the connection between the patient’s home to a VHA provider using streaming video. According to the Federal Communications Commission (FCC), video telemedicine requires minimum download/upload speeds of 25/3 Megabits per second (Mbps), ^14^ but depending on the number of devices simultaneously streaming content, latency, and other factors, higher download and upload speeds may be needed. Combined, these factors contribute to a “digital divide,” or the gap between those who do and do not have ready Internet availability and Internet-capable devices. VHA has attempted to bridge this divide by supplying patients Internet-connected devices, and negotiating with telecommunication companies to provide free unlimited data to veterans while using VHA video telemedicine,^15–17^ but such programs may be limited in rural or other inadequate broadband connectivity areas.^17^ Although the COVID-19 pandemic offered the opportunity to adopt new technologies, it also highlighted and potentially exacerbated existing care disparities, including between individuals with and without broadband speeds adequate for video-based telemedicine.^18^

In this study, we evaluate whether access to tele-MH services differed between veterans living in areas with optimal versus inadequate broadband speeds. We hypothesized that in the transition from in-person care to telemedicine at the onset of the COVID-19 pandemic, patients residing in inadequate broadband speed areas would have lower video telemedicine use. In these areas, we further hypothesized telephone care would supersede video and overall MH visits would be reduced. Identifying veterans with a measurable disparity in broadband availability may help evaluate the impact of unequal access to and use of tele-MH and inform health system policy initiatives to reduce the digital divide.

## METHODS

### Study Design

This is an instrumental variable difference-in-differences analysis using a retrospective cohort of VHA MH outpatient visits from October 1, 2015, to December 31, 2021. We followed the Strengthening the Reporting of Observational Studies in Epidemiology reporting guideline.^19^ The study was approved by the University of Iowa Institutional Review Board and the Iowa City VA Healthcare System Research and Development Committee. We conducted this work without direct patient contact using data routinely collected in the electronic health record and deemed of minimal risk; therefore, a waiver of informed consent was obtained.

### Data Sources

Data were obtained from the Veterans Informatics and Computing Infrastructure, an integrated system including all VHA electronic health records and administrative data. Patient-level data, including demographics, date, and outpatient delivery method, was obtained from the Corporate Data Warehouse. The 2010 Census Bureau TIGER/Line shapefile contains geographic entity codes. These data were spatially merged with the fiscal year-specific latitude and longitude of each Veteran’s home address to identify their census block group-based area deprivation index (ADI), a measure of neighborhood socioeconomic disadvantage^20^ and census block-based broadband availability using FCC Fixed Broadband data.^21^

### Patient Population

The study cohort included all veterans with an outpatient VHA MH visit between October 2015 and December 2021. Using 500-series stop codes, we included all in-person, telephone, or video-based MH encounters, as well as all specialty, non-specialty, and primary care-embedded MH encounters. We excluded care received at residential rehabilitation centers or nursing homes, and domiciliary care.

### Broadband Speed

Our primary exposure included census block broadband speeds at the patient’s residence, categorized as the availability of (1) inadequate (download  ≤25 Megabits per second - Mbps; upload  ≤3 Mbps), (2) adequate (download  ≥25 Mbps and  <100 Mbps; upload  ≥5 Mbps and  <100 Mbps), or (3) optimal (download and upload  ≥100/100 Mbps) broadband speeds. These speed thresholds are derived according to the FCC adequate speeds definitions,^22^ broadband technology speed capabilities, BroadbandNow quarterly reports,^23^ and senatorial calls to update broadband speed minimums to 100/100 Mbps.^24^ For each census block, the number of fixed Internet providers offering broadband at these speed thresholds was identified using December 2019 FCC Form 477.^21^ Broadband categories were assigned based on the highest broadband speed combination available, with at least one provider reporting such speeds. Fixed wireless and satellite Internet were excluded because these technologies, though available virtually everywhere, have low subscription rates, high costs, and less reliability compared to other broadband technologies.^25^ We note though FCC data is available biannually throughout our study period, variability in this definition over time was minimal.

### Outcomes

We assessed MH clinic visits by clinic stop codes and classified into mutually exclusive categories: in-person, telephone, or video-to-home via VA Video Connect. Once categorized, we calculated the number of MH visits per quarter by visit modality by patient and total MH visits per patient per quarter were enumerated. Visits were categorized as being pre- (October 2015–February 2020) or post-pandemic onset (March 2020–December 2021).

### Covariates

Patient demographics included age, sex, race, ethnicity, and patient rurality identified using the geocoded location of the patient’s home using Rural Urban Commuting Area codes and dichotomized into urban and rural (i.e., rural, highly rural, and insular categories).^26^ Race and ethnicity were self-reported. Race was categorized as Black or African American, other (including American Indian or Alaska Native, Asian, Multiracial, or Pacific Islander), and White. Ethnicity was reported as being Hispanic, Not Hispanic, or other (including missing, multiple ethnicities, or unknown).

### Statistical Analyses

We performed a difference-in-difference analysis evaluating the differences in MH visit count and MH visit modality in the period prior to and after onset of the COVID-19 pandemic. The dependent variable was a patient’s number of MH visits per quarter, by visit modality from October 2015 thru December 2021. Independent variables included a binary indicator for time before and after pandemic onset, a categorical variable of broadband speed category, and their interaction. The model was adjusted for patient characteristics, rurality, ADI, and quarter-year fixed effects using the Poisson model structure with Huber-White robust standard errors clustered at the census block. We report incidence rate ratios (IRRs). All hypothesis tests were two-sided with an a priori 0.05 level of significance. We plotted quarterly visit rates by modality and overall, from October 2017 to December 2021, as well as by broadband category. All analyses were conducted using SAS Enterprise Guide, version 8.2, and SQL Server Management, version 18.8.

Video visits are most impacted by broadband availability. Thus, we conducted subgroup analyses to determine whether there was varying association between video visit rates and broadband availability based on demographic factors by running the regression model for each demographic sub-group.

## RESULTS

During the 6-year study period, 3,659,699 unique veterans were seen by VHA MH clinicians, of which 87.9% were male; 66.6% White; 70.8% urban residing, with a mean age of 57.4 (SD = 16.9) years; 35.8% lived in an optimal broadband census block; 57.0% lived in an adequate broadband census block; and 7.2% lived in an inadequate census block (Table 1). Veterans living in census blocks with optimal versus inadequate broadband speeds were younger (mean [SD] age, 56.4 [16.9] years vs 60.2 [16.2] years), more likely to be Black (26.5% vs 12.5%), female (13.2% vs 9.5%), and live in an urban area (79.4% vs. 16.5%). More rural versus urban veterans (18.5% vs. 1.8%) live in an inadequate broadband census block (eTable 1). Comparatively, 22.6% of rural versus 42.2% of urban veterans live in areas with optimal broadband speeds.

**Table 1:** Study Cohort Demographics by Broadband Speed Category and Unadjusted Difference Comparing Optimal (Download / Upload ≥100/100 Mbps) and Inadequate (Download / Upload ≤25/3 Mbps) Broadband Speed Category Across Demographic Subgroups

Covariate	Inadequate^*^ broadband	Adequate^†^ broadband	Optimal^‡^ broadband	Unadjusted difference [inadequate vs. optimal] (95% CI)
Sample size, *N* (%)	264,401 (7.2)	2,084,903 (57.0)	1,310,395 (35.8)	
Age, mean (SD)	60.2 (16.2)	57.7 (16.9)	56.4 (16.9)	
*Female sex, N (%)*	25,010 (9.5)	234,832 (11.7)	173,362 (13.2)	3.8 (3.7 to 3.9)
*Race, N (%)*				
Black or African American	33,160 (12.5)	415,444 (19.9)	347,187 (26.5)	14.0 (13.8 to 14.1)
Other race^§^	9883 (3.7)	86,612 (4.2)	59,247 (4.5)	0.8 (0.7 to 0.9)
White	204,449 (77.3)	1,431,647 (68.7)	799,383 (61.0)	−16.3 (−16.5 to −16.1)
Unknown or missing	16,909 (6.4)	151,200 (7.3)	104,578 (8.0)	1.6 (1.5 to 1.7)
*Ethnicity, N (%)*				
Hispanic or Latino	12,920 (4.9)	172,446 (8.3)	117,949 (9.0)	4.1 (4.0 to 4.2)
Not Hispanic or Latino	238,267 (90.1)	1,801,166 (86.4)	1,119,195 (85.4)	−4.7 (−4.8 to −4.6)
Other^║^	13,214 (5.0)	111,291 (5.3)	73,251 (5.6)	0.6 (0.5 to 0.7)
Ever rural residence^¶^, *N* (%)	220,724 (83.5)	700,244 (33.6)	269,451 (20.6)	−62.9 (−63.1 to −62.8)
ADI^#^ national, mean (SD)	63.3 (22.9)	56.1 (25.3)	48.6 (26.0)	−14.8 (−14.9 to −14.7)

^*^Inadequate broadbands speeds are those  ≤25/3 Mbps

^†^Adequate broadbands speeds are those  ≥25/5 and  <100/100 Mbps

^‡^Optimal broadbands speeds are those  ≥100/100 Mbps

^§^Other race includes American Indian or Alaskan Native, Asian, Multiracial, or Pacific Islander categories

^║^Other ethnicity includes missing, more than 1, and unknown categories

^¶^Includes patients who lived in a rural area at any time during the study period

^#^*ADI* area deprivation index, ranks neighborhoods by socioeconomic disadvantage on a scale of 0–100 with lower rankings indicating less social disadvantage

**Figure 1:**
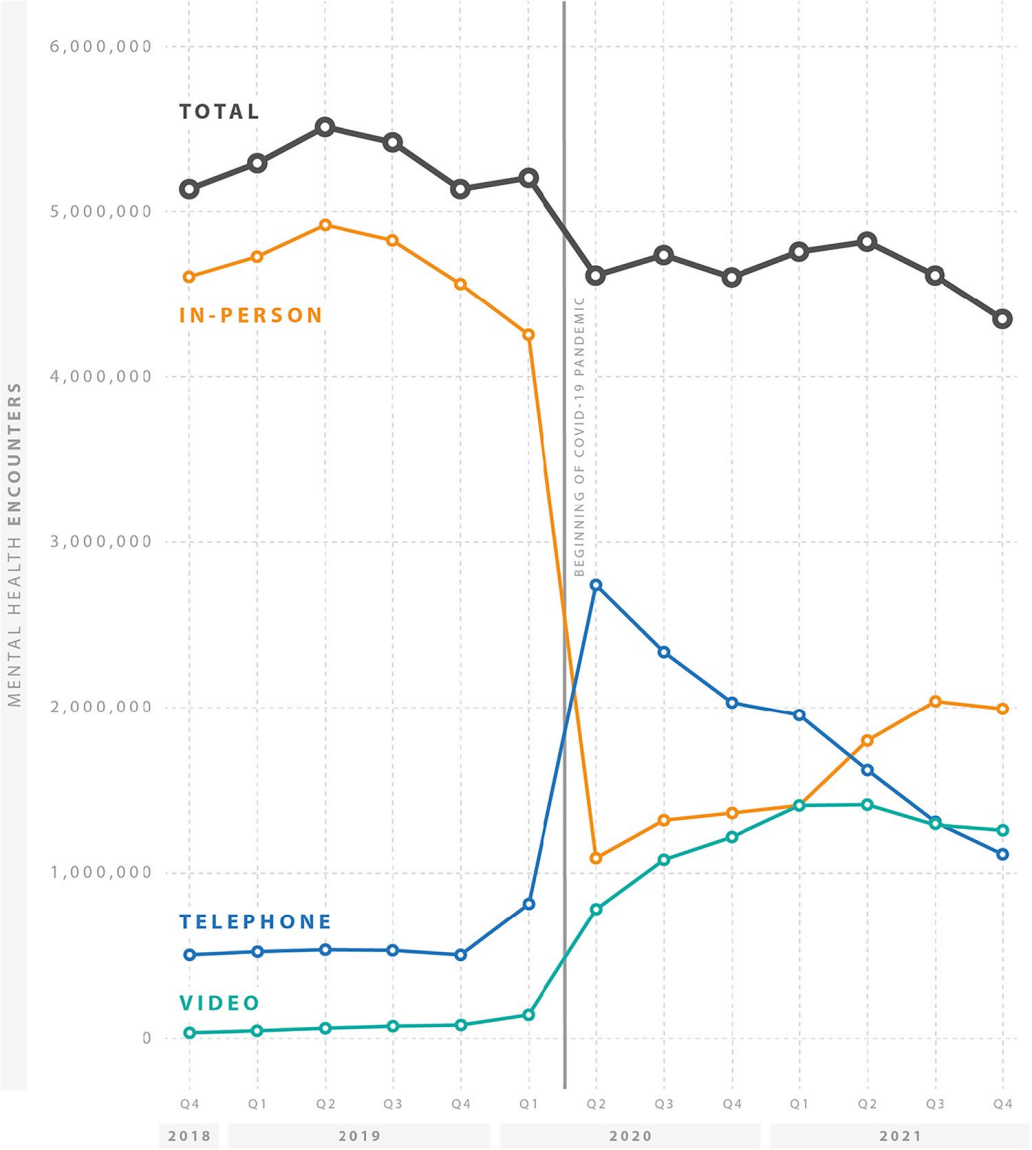
Mental health quarterly visit rate per 100 patients, total and by modality over time (October 2018–December 2021).

**Figure 2:**
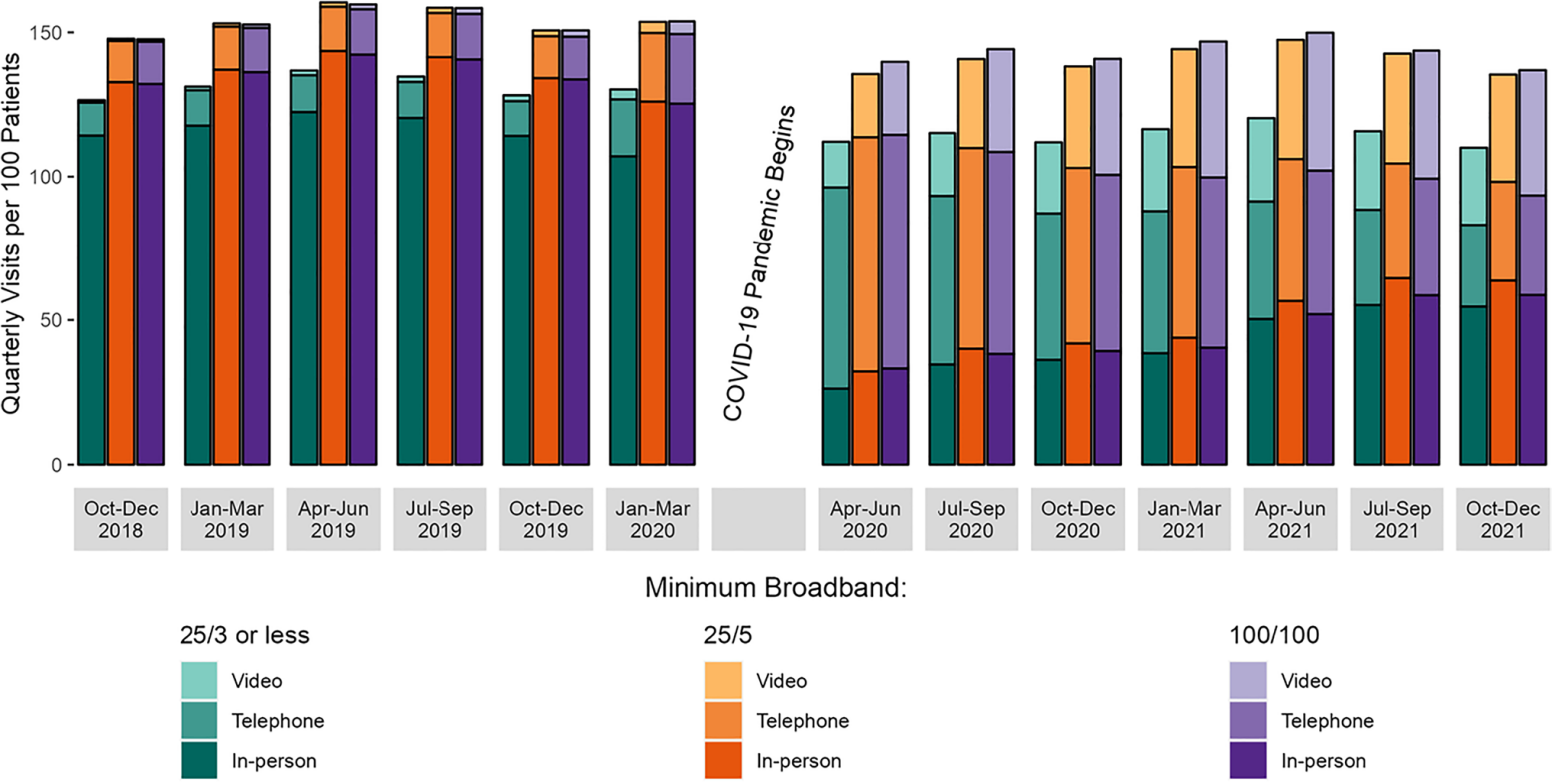
Mental health visits by modality by broadband speed categories.

Figure 1 illustrates the decline in MH visits overall after pandemic onset, in addition to the substitution of telephone and video visits. Although telephone was the predominant alternative modality to in-person visits, video visits continued to grow through much of 2021. By December 2021, MH visits were predominantly delivered in-person, though video and telephone visits remain a significant delivery modality. When considering broadband availability (Fig. 2), those residing in inadequate broadband areas display lower visit rates both before and during the pandemic than their more readily connected counterparts.

### Total Mental Health Visits

Pre-pandemic, total MH visits per 100 patients per quarter were lower for those in the inadequate, versus adequate or optimal, broadband speed categories (inadequate = 125.5 adequate = 149.3, and optimal = 149.2) (Table 2). After pandemic onset, total MH visits per 100 patients per quarter decreased regardless of broadband speed (inadequate = 114.5, adequate = 140.6, and optimal = 143.2). In adjusted analyses, patients living in optimal versus inadequate broadband census blocks experienced a smaller decline in total MH visit rate during vs. pre-pandemic (IRR = 1.05, 95% CI = 1.03–1.07).

**Table 2:** Mental Health Visit Rate Pre- and After Pandemic Onset, by Visit Type and Broadband Speed Category

Outcome (# of MH visits per 100 patients per quarter)	Inadequate* broadband (*N* = 268,804)	Adequate^†^ broadband (*N* = 2,118,282)	Optimal^‡^broadband (*N* = 1,331,803)
*In-person visits*			
Pre-COVID	113.3	133.9	132.9
COVID	42.2	48.9	45.7
IRR^§^ (95% CI)	*Reference category*	0.98 (0.96 to 1.00)	0.92 (0.90 to 0.94)
*Telephone visits*			
Pre-COVID	11.4	14.6	15.3
COVID	47.5	56.6	56.8
IRR (95% CI)	*Reference category*	0.93 (0.92 to 0.94)	0.89 (0.88 to 0.90)
*Video visits*			
Pre-COVID	0.9	0.8	0.9
COVID	24.9	35.1	40.6
IRR (95% CI)	*Reference category*	1.43 (1.37 to 1.50)	1.52 (1.45 to 1.59)
*Total visits of any type*			
Pre-COVID	125.5	149.3	149.2
COVID	114.5	140.6	143.2
IRR (95% CI)	*Reference category*	1.03 (1.02 to 1.05)	1.05 (1.03 to 1.07)

^*^Inadequate broadbands speeds are those  ≤25/3 Mbps

^†^Adequate broadbands speeds are those  ≥25/5 and  <100/100 Mbps

^‡^Optimal broadbands speeds are those  ≥100/100 Mbps

^§^IRR (incident rate ratio) is based on a Poisson regression model that estimated the change pre- versus after the onset of the pandemic in patients’ quarterly count of MH visits by type among patients living in census blocks with 25/5 Mbps or 100/100 Mbps broadband, compared to the change among patients living in census blocks with 25/3 Mbps or less broadband, with adjustment for patient and geographic covariates and quarter-year fixed effects

### Video Mental Health Visits

Video MH visits per 100 patients per quarter were similar across all broadband speed categories in the pre-pandemic period (inadequate = 0.8, adequate = 0.8, and optimal = 0.9) and universally higher after pandemic onset (inadequate = 24.9, adequate = 35.1, and optimal = 40.6). In adjusted analyses, patients living in optimal versus inadequate broadband census blocks exhibited a greater increase in the rate of video MH visits post- versus pre-pandemic (IRR = 1.52 95% CI = 1.45–1.59); increases were less but still significantly different when comparing adequate versus inadequate broadband categories (IRR = 1.43, 95% CI = 1.37–1.50).

### Telephone Mental Health Visits

Telephone MH visits per 100 patients per quarter varied based on broadband speeds pre-pandemic (inadequate = 11.4, adequate = 14.6, and optimal = 15.3) and were higher in all categories after pandemic onset (inadequate = 47.5, adequate = 56.6, and optimal = 56.8). In adjusted analyses, patients living in optimal versus inadequate broadband census blocks experienced a smaller increase in the rate of telephone MH visits over the study period (IRR = 0.89, 95% CI = 0.88–0.90; *P* < 0.001); decreases were similar for adequate versus inadequate broadband categories (IRR = 0.93, 95% CI = 0.92–0.94; *P* < 0.001).

### Subgroup Analysis

In sub-group analysis, video visits increased across all patient characteristics during the COVID-19 pandemic (Table 3). The largest gains in video visit rates per 100 patients per quarter were among those in optimal broadband census blocks, especially among younger (age 18–43 years: difference 51.8), female (difference 67.3), and Hispanic (difference 47.3) veterans. When evaluating the association with ADI, those in the lowest quartile (indicating least social disadvantage) had the largest gains in video visit rates across all ADI categories. In adjusted analyses, patients in the lowest ADI category living in optimal versus inadequate broadband census blocks exhibited an increase in video visit rates after versus before pandemic onset (IRR = 1.4, 95% CI: 1.2 to 1.7) as did patients in the lowest ADI category living in adequate versus inadequate broadband census blocks (IRR = 1.2, 95% CI: 1.0 to 1.5).

**Table 3:** Association Between Patient Demographics and the Proportion of Quarterly Video Mental Health Visit Rate per 100 Patients

	Number of MH video visits per 100 patients per quarter			Patients with adequate* availability compared to inadequate^†^ availability	Patients with optimal^‡^ availability compared to inadequate availability
Covariate	Inadequate availability	Adequate availability	Optimal availability		
	Post minus pre-COVID-19 pandemic mean difference			IRR (95% CI)	
*Age (years)*					
Quartile 1 (18–43)	38.9	47.7	51.8	1.3 (1.2 to 1.4)	1.4 (1.3 to 1.5)
Quartile 2 (44–60)	34.2	45.1	49.3	1.4 (1.3 to 1.6)	1.5 (1.4 to 1.6)
Quartile 3 (61–71)	16.7	25.0	29.8	1.6 (1.4 to 1.8)	1.9 (1.7 to 2.1)
Quartile 4 (≥72)	8.6	12.7	16.7	1.7 (1.5 to 1.9)	2.0 (1.7 to 2.3)
*Sex*					
Female	48.4	62.2	67.3	1.4 (1.3 to 1.6)	1.5 (1.3 to 1.6)
Male	21.2	30.2	35.1	1.4 (1.4 to 1.5)	1.6 (1.5 to 1.6)
*Race*					
Black or African American	28.2	36.9	42.8	1.5 (1.3 to 1.6)	1.4 (1.3 to 1.6)
Other race^§^	29.4	39.8	47.9	1.3 (1.1 to 1.6)	1.3 (1.1 to 1.6)
White	23.4	33.5	37.9	1.4 (1.3 to 1.5)	1.5 (1.4 to 1.6)
Missing	19.8	31.1	35.7	- -	- -
*Ethnicity*					
Hispanic or Latino	33.0	40.5	47.3	1.3 (1.1 to 1.5)	1.2 (1.1 to 1.5)
Not Hispanic or Latino	23.6	33.8	39.2	1.4 (1.4 to 1.5)	1.5 (1.5 to 1.6)
Other^║^	22.5	31.2	34.8	- -	- -
*Area Deprivation Index*^*¶*^					
Quartile 1 (0–25)	36.4	42.7	47.8	1.2 (1.0 to 1.5)	1.4 (1.2 to 1.7)
Quartile 2 (26–50)	27.3	37.1	41.6	1.4 (1.3 to 1.5)	1.5 (1.4 to 1.6)
Quartile 3 (51–75)	23.6	32.4	36.7	1.4 (1.3 to 1.5)	1.4 (1.3 to 1.5)
Quartile 4 (76–100)	20.0	28.4	31.1	1.5 (1.4 to 1.7)	1.5 (1.4 to 1.6)

^*^Adequate broadbands speeds are those  ≥25/5 Mbps and  <100/100 Mbps

^†^Inadequate broadbands speeds are those  ≤25/3 Mbps

^‡^Optimal broadbands speeds are those  ≥100/100 Mbps

^§^Other race includes American Indian or Alaskan Native, Asian, Multiracial, or Pacific Islander categories

^║^Other ethnicity includes missing, more than 1, and unknown categories

^¶^*ADI* area deprivation index, ranks neighborhoods by socioeconomic disadvantage on a scale of 0–100 with higher rankings indicating more social disadvantage

## DISCUSSION

The COVID-19 pandemic required a rapid transition from in-person visits to telemedicine. We examined whether this adjustment was associated with MH utilization among veterans by broadband speeds. Overall, MH visits have not recovered to pre-pandemic levels regardless of broadband category. For veterans living in inadequate broadband service areas, MH visit rates were significantly lower before the pandemic began. This disparity widened as in-person visits were restricted, despite the growth of telephone and video visits. With VHA’s continued commitment to provide care where and how veterans need it, and the amenability of MH services to telemedicine,^4,8^ these results underscore the importance of optimal broadband connectivity for improved MH access. Importantly, unequal broadband connectivity can exacerbate existing disparities, for example among rural populations, limiting tele-mental access, and further fragmenting care. Coupled with other barriers such as transportation or caregiving responsibilities, this may result in an inability to access timely care altogether. Given predictions of long-term COVID-19 MH impacts, expanding broadband coverage (a social determinant of health itself)^27^ and developing innovative telemedicine solutions should be considered.

Telephone-based visits increased in all groups after pandemic onset. Those with optimal (or adequate) versus inadequate broadband were less likely to use telephone, aligning with recent research.^28^ In many treatment contexts, MH services delivered by video or telephone have been noninferior to in-person care.^4^ Because visual cues may provide useful clinical information and improve patient-clinician rapport among other benefits, access to video-based care becomes a quality-of-care issue. Nonetheless, telephone-based access is preferable to no access and may even be favored in some instances. For example, in a study of 25 primary care, MH, and pediatric clinicians in community health centers, Chang et al.^29^ reported although MH clinicians acknowledged issues reading body language and facial expressions during telephone psychotherapy visits, telephone allowed greater privacy and at times increased sharing. Although most patients preferred video-based encounters, some preferred telephone underscoring the importance of patient preferences when evaluating treatment delivery options.^29^

VHA has invested considerable resources in tele-MH services and developed a digital divide consult to overcome infrastructure-related barriers, including tablet distribution to eligible veterans and resources for discounted Internet access^30^. These programs represent valuable alternatives while residential broadband expands in areas of greatest need. These include Clinical Resource Hubs (CRH), which use team-based, technology-facilitated care to increase access to primary care, MH, clinical pharmacy, and other specialties;^31^ MH CRHs were established in 2019 in all 18 VHA Veterans Integrated Service Networks.^32^ These “hubs” serve medically underserved “spoke” sites through telemedicine or in-person care, allowing veterans to receive care at their local VA clinic if broadband adequacy precludes home tele-MH encounters. In over 450 sites nationally, CRHs served over 680,000 veterans between September 2019 and September 2020.^33^

These results should be considered specifically among rural populations, who often live in MH professional shortage areas^34^ and experience larger unadjusted suicide rates.^35^ In previous research, although video-based MH care increased for rural and urban populations, rural veterans received a smaller proportion of their care via video during the pandemic.^36,37^ However, a recent study found rural versus urban residents were more interested in tele-MH services^38^ and take advantage of mental health services as they become available^39^ underscoring the need for educational and exposure opportunities (i.e., digital skills and literary programs) for telemedicine at key points in the access trajectory.^40^ According to our study, despite improvements,^41,42^ this may be due in part to broadband coverage limitations, which are more prevalent among rural populations and will not be quickly resolved. To overcome this issue, VHA could expand clinical video telehealth, which connects a veteran at a VHA community-based clinic to a provider elsewhere. This may require a continuation of the clinic-based broadband upgrades that took place in 2018–2021. Additionally, as eligible veterans obtain community-based care thru VHA due to drive time and access standards^43^, opportunities for collaboration across healthcare systems extending tele-MH access may exist. In a recent pilot study, eligible rural veterans were connected to VHA telemedicine appointments following clinic appointments at federally qualified health centers, mitigating issues with home broadband connectivity.^44,45^ The VHA should also work with infrastructure partners, like the FCC and telecommunications companies, to prioritize broadband Internet development based on the greatest community-level impact.

This research has limitations. First, we did not account for patient comorbidities, clinician familiarity, or other factors affecting telemedicine demand including digital literacy or socioeconomic status. Second, we were unable to distinguish when a video visit reverted to telephone thereby possibly overcounting video visits when broadband was inadequate. Understanding the technology barriers affecting video-based telemedicine should be considered. Finally, FCC data inadequacies, including our inability to jointly account for cellular service, which is reported by technology types (versus speeds) and coverage areas (instead of census blocks) and imprecise reporting standards (e.g., providers need only offer service to one location within a census block), may affect the estimation of broadband availability. We acknowledge individual barriers may exist to accessing the maximum broadband speed available within the census block, including cost, infrastructure changes, veteran preferences, digital literary, or other reasons. Despite these limitations, this work is the first we are aware of using FCC data by varying speed thresholds to triangulate the impact of broadband availability and disparities in VHA tele-MH services.

The COVID-19 pandemic dramatically expanded tele-MH in VHA. Though MH services are well-suited to video-based telemedicine, disparities based on broadband availability are a growing equity concern. This study adds to the growing literature about MH visits, video-based telemedicine, and the impact of broadband availability on both.

## Supplementary Information

Below is the link to the electronic supplementary material.

Supplementary file1 (DOCX 40 KB)

## Data Availability

The datasets generated and analyzed are not publicly available due to VHA privacy and confidentiality requirements but are available to VHA researchers from the corresponding author on reasonable request.
