# Supplementary material for: Quantifying the Digital Divide: Associations of Broadband Internet with Tele-mental Health Access Before and During the COVID-19 Pandemic
Source: J Gen Intern Med. 2023 Jun 20;38(Suppl 3):832–40. doi: 10.1007/s11606-023-08120-8 (PMC10356688; doi:10.1007/s11606-023-08120-8)
Supplement: Supplementary file 1 — Supplementary file1 (DOCX 40 KB) [file 11606_2023_8120_MOESM1_ESM.docx]

**Appendix**

**A. The Primary Statistical Model**

The primary statical model is:

y_it_ = α_1_*post_t +_* α_2_*broadband_i +_* α_3_*post_t_ * broadband_i_* + α_4_*X_i_* + α_5_*ADI_c_* + α_6_*rural_c_* + σ_t_+ ε_c_

The dependent variable (y_it_) was patient *i*'s number of mental health visits per quarter *t*, by visit modality. Independent variables included a binary indicator for time before and after pandemic onset (*post_t_*), a categorical variable for each broadband speed category (*broadband_i_*), and their interaction. The model was adjusted for a vector of patient characteristics (*X_i_*) including age (years), binary indicators for female, Black, and Hispanic, the area deprivation index (*ADI_c_*) of the census block (*c*), the rurality of the census block (*rural_c_*), and quarter-year fixed effects (σ_t_). The coefficient of interest was α_3_.

**eTable1. Broadband Category by Patient Rurality**

| **Broadband Category** | **Rural**  **(N=1,190,419)** | **Urban**  **(N=2,469,280)** |
| --- | --- | --- |
| **Inadequate*** | 220,724 (18.54) | 43,677 (1.77) |
| **Adequate**^†^ | 700,244 (58.82) | 1,384,659(56.08) |
| **Optimal**^‡^ | 269,451 (22.63) | 1,040,944 (42.16) |

* Inadequate broadbands speeds are those ≤25/3 MB/s

† Adequate broadbands speeds are those ≥25/5 and <100/100 MB/s

‡ Optimal broadbands speeds are those >100/100 MB/s
